# Morphological Analysis of Human Milk Membrane Enclosed Structures Reveals Diverse Cells and Cell-like Milk Fat Globules

**DOI:** 10.1007/s10911-020-09472-1

**Published:** 2021-01-04

**Authors:** Isabel Schultz-Pernice, Lisa K. Engelbrecht, Stefania Petricca, Christina H. Scheel, Alecia-Jane Twigger

**Affiliations:** 1 Helmholtz Zentrum München, German Research Center for Environmental Health, Institute of Stem Cell Research, Ingolstädter Landstrasse 1, 85764 Neuherberg, Germany; 2 Department of Dermatology, Ruhr-University Bochum, North Rhine-Westphalia, Bochum, Germany

**Keywords:** Human milk cells, Milk fat globules, Human lactation, Membrane enclosed structures, Human milk, Binucleated cells, Secretory luminal cells

## Abstract

Over the past decade, the cellular content of human milk has been a focus in lactation research due to the benefit a potential non-invasive stem cell compartment could provide either to the infant or for therapeutic applications. Despite an increase in the number of studies in this field, fundamental knowledge in regard to milk cell identification and characterisation is still lacking. In this project, we investigated the nature, morphology and content of membrane enclosed structures (MESs) and explored different methods to enrich human milk cells (HMCs) whilst reducing milk fat globule (MFG) content. Using both flow cytometry and immunofluorescence imaging, we confirmed previous reports and showed that nucleated HMCs make up a minority of milk-isolated MESs and are indistinguishable from MFGs without the use of a nuclear stain. HMC heterogeneity was demonstrated by differential uptake of nuclear stains Hoechst 33258 and DRAQ5™ using a novel technique of imaging milk MESs (by embedding them in agar), that enabled examination of both extracellular and intracellular markers. We found that MESs often contain multiple lipid droplets of various sizes and for the first time report that late post-partum human milk contains secretory luminal binucleated cells found across a number of participants. After investigation of different techniques, we found that viably freezing milk cells is an easy and effective method to substantially reduce MFG content of samples. Alternatively, milk MESs can be filtered using a MACS® filter and return a highly viable, though reduced population of milk cells. Using the techniques and findings we’ve developed in this study; future research may focus on further characterising HMCs and the functional secretory mammary epithelium during lactation.

## Background

Breastfeeding provides rich and adaptive milk constituents to the developing infant promoting brain development and cognitive functioning as well as reducing the lifetime risk for the infant acquiring diseases such as diabetes and obesity [1–3]. In addition to the more thoroughly investigated bioactive milk constituents such as fatty acids, carbohydrates, proteins, growth factors, cytokines and bacteria [4–6], less studied components such as human milk cells (HMCs) may provide an undiscovered benefit to the breastfeeding dyad.

Human milk has been reported to contain between 1 × 10^4^ and 13.5 × 10^6^ eukaryotic cells/mL of milk, consisting of a range of different immune, epithelial and progenitor cell types [7]. As the number of eukaryotic cells per mL of milk varies between publications, recent studies suggest that factors such as stage of lactation, time of day, time since last feed and health status of the mother/infant dyad may impact the number of cells and sub-population frequencies observed across different samples [7–9]. Intriguingly, studies suggest that a proportion of this cell fraction might survive the passage of the infant’s gastrointestinal tract and may play an important role in providing benefits to the breastfeeding new born [5, 10–13]. Based on these findings, human clinical trials have been initiated to investigate the potential beneficial effects of administrating human milk through the intranasal cavity of tube-fed pre-term infants [14].

Together with earlier publications [15, 16], a recent study by Keller et al. [17] suggests that only a minority of pelleted structures obtained by centrifugation of human milk are in fact nucleated. On average only 9% (range 0.7–63%) of recorded events were found to stain positive for nuclear dye DRAQ5™ and of these 8 to 81% represent viable cells that excluded dead cell stain Sytox™ Blue [17]. Remarkably, it was found that a majority of structures isolated from human milk are non-nucleated particles that absorb neutral lipid stain Nile red [17] suggesting they likely represent milk fat globules (MFGs).

MFGs in human milk have been reported to vary in size (range of 0.35–13 μm), being membrane enclosed and contain a single triglyceride core intermittently surrounded by a cytoplasmic remnant [18, 19]. Indeed, previous literature examining either cells [7, 16, 20] or fat globules [15, 16, 18, 19, 21] isolated from milk suggest that both are structurally distinct, although direct morphological comparisons are scarce. Interestingly, forward scatter/side scatter (FSC/SSC) dot plots presented by Keller et al. [17] suggest that DRAQ5™^+^ nucleated and Nile red^+^ lipid-containing structures isolated from human milk are of similar sizes and granularity although both dyes were never used to directly co-stain samples. Based on this data, there may be potential similarities between MFGs and HMCs that, representing the main part of milk pelleted structures and being both surrounded by membranes, will be collectively referred to as membrane enclosed structures (MESs) from this point on. What is also unclear is whether DRAQ5™ is able to penetrate all milk cell types or whether DRAQ5™ and Nile red commonly co-stain within the same MES. The aim of this study was to compare the morphology of HMCs and MFGs to better understand, distinguish and exclude non-nucleated MFGs from downstream cellular analysis.

Here, we combine the nuclear stain DRAQ5™ with neutral lipid stain Nile red together with nuclear stain Hoechst 33258 to investigate HMC and MFG composition and morphology using both flow cytometry and our adapted technique of agarpad immunofluorescence imaging. Firstly, we confirm previous findings that the pelleted fraction of human milk is dominated by MFGs but go further to find similarities in the morphology of HMCs compared to MFGs. Both MFGs and HMCs may contain multiple lipid droplets and we find heterogeneity in milk cell morphology, with a subset being binucleated. Additionally, we examine strategies to enrich HMCs whilst reducing the number of MFGs within the pelleted fraction of human milk using viable freezing and MACS® dead cell removal.

## Methods

### Human Milk Sample Collection

Human milk samples for this study were collected according to an ethics vote approved by the Ludwig-Maximilians-Universität München ethics committee (project number: 17–715). Breastfeeding participants (*n* = 27) provided written informed consent, demographic information via a voluntary questionnaire (Table 1), together with one or more fresh milk samples (7,5–113,5 mL, *n* = 41). We found no significant differences between the age (*P* = 0.5), parity (*P* = 0.7), infant’s gestational age (*P* = 0.8) and infant’s age at collection (*P* = 0.08) of participants whose milk samples were used for either microscopic or flow cytometry analysis. Samples were collected under aseptic conditions using either a double electric breast pump (Symphony; Medela AG, Baar, Switzerland) or participant’s personal pump. Milk collections were obtained either at the participant’s homes, postpartum educational classes or within the lactation room at the Helmholtz Center Munich, depending on the preferences of the participant. After collection, all milk samples were transported immediately on ice and processed as soon as possible (< 2 h after collection).

**Table 1:** Demographics of study participants

	**All Participants**	**Microscopy Participants**	**Flow Cytometry Analysis Participants**
	n = 27	*n* = 27	*n* = 18
Age of donor (years)	36 (26–43)*	36 (26–43)*	35 (26–43)
Parity	2 (1–3)*	2 (1–3)*	2 (1–3)
Infant gestational age (weeks)	39 (34–41)*	39 (34–41)*	39 (34–41)*
Infant age at collection (weeks)	28 (3–62)*	28 (3–62)*	20 (3–61)
Milk volume (mL)	53 (7,5–113,5)	53 (7,5–113,5)	47 (17–92,5)

*indicates not all donors were included due to missing information

### Human Milk Membrane Enclosed Structure Isolation

Human MESs were isolated by diluting milk samples in an equal volume of sterile phosphate–buffered saline (PBS) (ThermoFisher Scientific, Waltham, U.S.) and centrifuging at 870 g for 20 min at 20 °C in a Rotanta 460R centrifuge (Hettich, Tuttlingen, Germany). The pellet was washed by removing the supernatant and resuspending in 5 to 10 mL of cold PBS before transferring the sample to a new 15 mL tube (Corning, Corning, U.S.) and centrifuging at 490 g for 5 min at 4 °C. Following a second washing step, 100 to 550 μL of mammary epithelial cell growth medium (MECGM) (PromoCell, Heidelberg, Germany) was added to the MES aggregations according to the pellet size. Subsequently, cells were either frozen or used immediately for downstream analysis.

### Freezing and Thawing Procedure of Isolated Human Milk Membrane Enclosed Structures

Freezing medium was prepared by mixing 40% MECGM with 50% fetal calf serum (FCS) (PAN-Biotech, Aidenbach, Germany) and 10% dimethyl sulfoxide (DMSO) (Merck, Darmstadt, Germany). Cells were counted using a Neubauer Improved counting chamber (Omnilab, Vienna, Austria). Subsequently, freezing medium was added to the cell pellet at a concentration of approximately 5 × 10^6^ MESs/mL. MES suspensions containing cells were rapidly transferred into CryoTube™ vials (Corning, Corning, U.S.) and immediately frozen at −80 °C. Vials were later transferred into liquid nitrogen tanks for long term storage.

When required, frozen MESs were thawed in a 37 °C pre-heated water bath (Memmert, Schwabach, Germany) and immediately resuspended in 15 mL MECGM. Cells were pelleted by centrifugation at 490 g for 5 min at 4 °C and resuspended in ~150 μL of MECGM.

### Immunofluorescence Staining of Human Milk Membrane Enclosed Structures

To discriminate and quantify MFGs and HMCs in the freshly isolated/post-freezing MES pellet, bright field and immunofluorescence microscopy was performed. 100 μL of MESs suspended in MECGM or PBS were incubated with Hoechst 33258 (final concentration 1 μg/ml) (H1398, ThermoFisher Scientific, Waltham, U.S.) at room temperature for 20 min in the dark. Subsequently, DRAQ5™ (62,254, ThermoFisher Scientific, Waltham, U.S.) and Nile red (N3013-100MG, Merck, Darmstadt, Germany) were added to a final concentration of 0.4 μg/mL (1 mM) and 0.1 μg/mL respectively and the MESs incubated for a further 5 min in the dark. Stained MESs were either added to a Neubauer Improved counting chamber for counting, imbedded in agarpads (see agarpad mounting) prior to imaging or analysed using flow cytometry (see flow cytometry analysis).

Agarpad analysis of cells was additionally performed to analyse CD36, Mucin-1 and cytokeratin 8/18 (CK8/18) presence and distribution. MESs were fixed for 15 min with 700 μl of a 4% paraformaldehyde (PFA) (ThermoFisher Scientific, Waltham, U.S.) solution and subsequently washed by adding 1000 μl fresh PBS. Following centrifugation at 400 g for 5 min at 4 °C, the pelleted MESs were permeabilized by adding a 0.1% Triton X-100 (Merck, Darmstadt, Germany) in PBS solution and incubating 15 min at room temperature. Permeabilized MESs were washed once more as described above and resuspended in staining solution which was specific to the antibodies used. Firstly 100 μl of 1:40 CD36-PE conjugated antibody (cat number: 555455, BD Pharmingen, Allschwil, Switzerland) and 0.42:100 Mucin-1-FITC conjugated antibody (CD227 (MUC1) FITC, cat number: 559774, BD Pharmingen, Allschwil, Switzerland) was added to the MESs and incubated at room temperature in the dark for 45 min. Alternatively, 500 μl of a 1:100 primary CK8/18 antibody (DLN-010750, Dianova, Castelldefels, Spain) in blocking buffer (PBS 0.1% BSA (Merck, Darmstadt, Germany) with 10% normal donkey serum (GeneTex, Irvine, U.S.)) solution for 3 h at room temperature. MESs stained with CK 8/18 were then washed again and resuspended in 500 μl of a 1:250 secondary antibody in (anti-mouse Alexa Fluor 488 Donkey Anti-Mouse IgG, A21202, ThermoFisher Scientific, Waltham, U.S.) PBS 0.1% BSA solution and incubated for further 45 min at room temperature. After antibody incubation, 500 μL of a 0.4 μg/mL DAPI solution (D9542-1MG, Merck, Darmstadt, Germany) were added to the MES suspension (stained with either CD36/Mucin-1 or CK 8/18) and incubated for 2 min. MESs were washed and embedded into an agarpad (see agarpad mounting).

### Agarpad Mounting of Milk MESs

For high resolution imaging and long-term storage of cell slides, cells were embedded into thin sheets of agar (agarpads) upon staining. Hoechst33258/DRAQ5™/Nile red stained milk MESs and primary HMECs were diluted in PBS, pelleted by centrifugation (400 g, 5 min, 4 °C) in a Heraeus Fresco 21 microcentrifuge (ThermoFisher Scientific, Waltham, U.S.) and fixed for 10 min with 500 μL of 4% PFA solution. MESs were then washed by adding 500 μL of PBS to the PFA solution, pelleted by centrifugation (400 g, 5 min, 4 °C). Subsequently approximately 0.3 × 10^6^–0.5 × 10^6^ MESs were resuspended in 25–20 μL of 30 °C 0.5% agarose (VWR, Radnor, U.S.) in PBS and pipetted onto a glass slide (Hecht, Sondheim von der Rhön, Germany) being careful not to introduce bubbles into the mixture. The embedded MESs were then rapidly topped with a 22 mm × 22 mm coverslip (VWR, Radnor, U.S.) and sealed with nail polish after drying.

### Counting Isolated Human Milk Cells and Milk Fat Globules

To assess the number of HMCs and MFGs per mL of milk, the number of MESs present on each image were counted and categorized according to their staining. The total number of HMCs was defined as the number of MESs staining positive for DRAQ5™ and/or Hoechst 33258, while MFGs showed Nile red staining only. The number of MESs per mL was subsequently calculated by multiplying the number of MESs per image with the dilution factor (DF) and dividing the product by the number of big squares counted and milk volume of the sample: $\frac{n\  cells\ast DF\ast {10}^4}{n\  squares\ast mL\  milk}$ .

### Flow Cytometry

Flow cytometry was employed to examine MES size and granularity together with nuclear or neutral lipid dye absorption. MESs were stained either with DRAQ5™, Hoechst 33258 and Nile red as described above, or with DRAQ5™ and dead cell stain SYTOX™ Blue (ThermoFisher Scientific, Waltham, U.S.) (final concentration 1 μM/100 μL). After incubation, stained MESs were diluted in MECGM and filtered through 35 μm cell strainer caps of round-bottom tubes (Corning, Corning, U.S.). Flow cytometry was performed using a FACSAria™ III cell sorter (BD Biosciences, Franklin Lakes, U.S.) with a 100 nm nozzle in combination with FACSDiva™ 6.0 Software. Laser settings were adjusted using unstained and single stain controls. Obtained data was analysed using the FlowJo_V10 Software (FlowJo LLC, Ashland, U.S.).

### Live Cell Isolation by Dead Cell Removal

To assess whether HMCs could be separated from cell fragments, debris and MFGs by filtration, MACS® Dead Cell Removal Kit technology (Miltenyi Biotec, Bergisch Gladbach, Germany) was employed to filter fresh or frozen MESs. First, MESs were pelleted by centrifugation at 300 g for 5 min at 4 °C. The pellet was resuspended in 100 μL of Dead Cell Removal MicroBeads. Following an incubation period of 15 min, 500 μL of binding buffer, diluted according to the protocol provided by the supplier, were added and the suspension applied to a MACS® column, previously rinsed with 500 μL binding buffer and attached to the magnetic field of a MACS® separator. Subsequently, the column was washed 4 more times with binding buffer. The collected live cell suspension was centrifuged at 4 °C for 5 min at 400 g, the buffer discarded and the cells resuspended in 50 to 100 μL MECGM.

### Image Acquisition and Processing

Bright field images were acquired using a Leica DM IL LED microscope (Leica Biosystems, Wetzlar, Germany) and immunofluorescence images were taken on an Axio Imager M2 microscope (Zeiss, Oberkochen, Germany) with Zeiss ZEN 2.3 pro software, 20x and 63x objectives and 385 nm, 567 nm and 630 nm LED modules. Imaging post processing was conducted using the open source software GIMP 2.8.22.

### Statistics

Graphs were generated using R version 3.6.2 with packages ggplot2 and dplyr (for data arrangement) [22–24]. T-test analysis was performed using Excel (version 2003) and applied to compare two groups of data. *P* values <0.05 were considered to be significant.

## Results

### Immunofluorescence Microscopy Confirms Large Numbers of Milk Fat Globules amongst Freshly Isolated Human Milk Cells

**Fig. 1:**
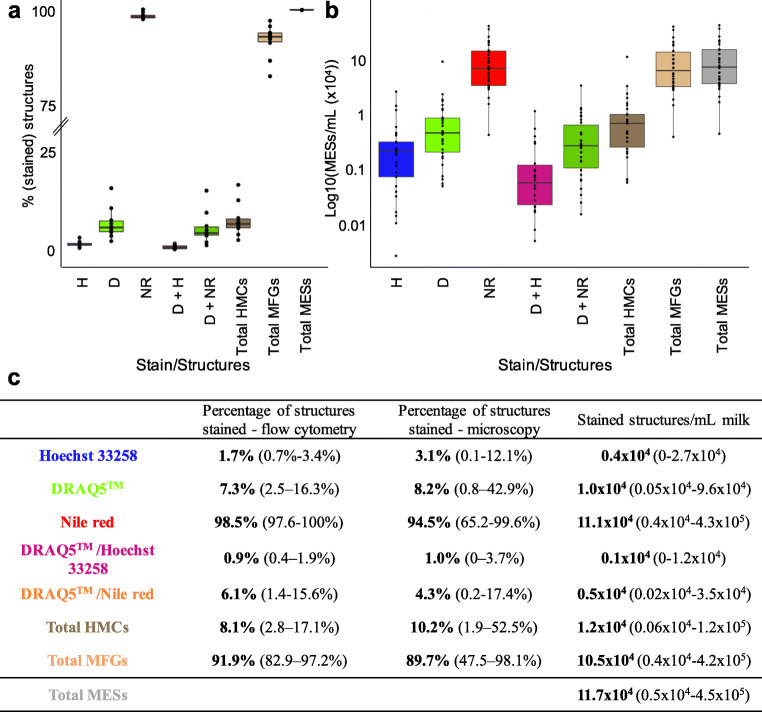
**Membrane enclosed structure (MES) staining quantification**. A) Percentage of stained MESs per single stain or stain combination and structure types according to flow cytometry. B) Logarithmic representation of number of stained MESs per mL of milk (×104) according to microscopic analysis. C) Percentage, estimated number and range of human milk cells (HMCs), milk fat globules (MFGs) and stained structures in total MES content as determined by Hoechst 33258, DRAQ5^TM^ and Nile red staining and detected either with flow cytometry or immunofluorescence microscopy. Abbreviations: H: Hoechst 33258; D: DRAQ5^TM^; NR: Nile red.

**Fig. 2:**
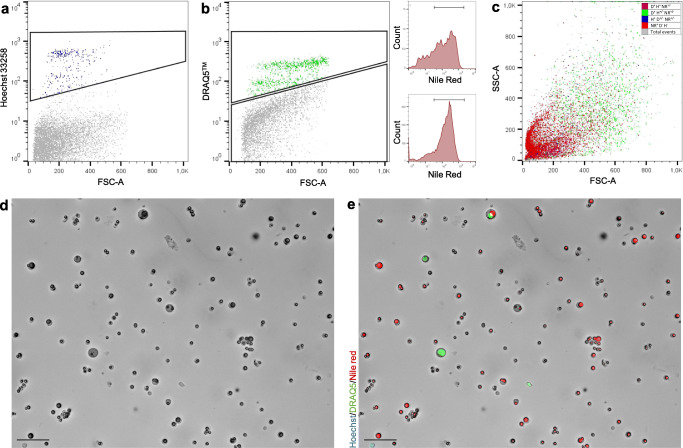
**Flow cytometry and microscopical analysis of Hoechst 33258, DRAQ5**^**TM**^ **and Nile red stained membrane enclosed structures (MESs).** A) MESs positively staining for Hoechst 33258 (blue). B) MESs positively staining for DRAQ5^TM^ (green). Side graphs show Nile red was detected both in DRAQ5^TM^+ and DRAQ5^TM^- structures. C) MESs distribution according to granularity (SSC-A) and size (FSC-A). D) Bright-field image of MESs, unstained. E) MESs stained with Hoechst 33258, DRAQ5^TM^ and Nile red in the same field of view as D. Scale bars: 50 μm. Abbreviations: H: Hoechst 33258; D: DRAQ5^TM^; NR: Nile red

Whilst past publications may have considered all MESs in the pelleted fraction of milk as cells, our data corroborates recent findings suggesting that merely 9% of all pelleted MESs are HMCs [17]. Indeed, using both flow cytometry and fluorescence microscopy, we found that whilst a majority of MESs isolated from human milk absorb lipid stain Nile red, only a small subset are nucleated and incorporate DNA stains DRAQ5™ and/or Hoechst 33258 (Fig. 1**,** Fig. 2). Strikingly, flow cytometry revealed that Hoechst 33258 stained significantly less MESs than DRAQ5™ (1.7% of total MESs compared to 7.3% of total MESs, *P* = 0.007) where only 0.9% of MESs stained positive for both nuclear stains (Fig. 1A, C). Differential incorporation of these dyes suggests milk cell heterogeneity and demonstrates that DRAQ5™ is able to integrate into a greater number of HMCs. Whilst a majority of non-nucleated MESs absorbed Nile red (Fig. 1, Fig. 2B), we found that 6.1% of DRAQ5™^+^ MESs also absorbed high levels of Nile red (Fig. 1A, C, Fig. 2B) indicating nucleated structures often also contain lipid droplets. As was previously suggested (but not confirmed by co-staining of DRAQ5™ and Nile red [17]), nucleated HMCs and non-nucleated lipid containing MFGs display similar size and granularity (as indicated by the forward FSC-A and side scatter SSC-A, Fig. 2C).

Results obtained by flow cytometry were corroborated by loading stained MESs onto a counting chamber, visualizing with a fluorescence microscope and quantifying stained structures (Fig. 1B, C). Without the use of the nuclear stains, HMCs and MFGs appear to be of similar sizes and are visually indistinguishable by eye (Fig. 2D, E). Confirming previous observations [7–9] we documented wide daily variation within participants for total MES content (average difference between donations: 13.4 × 10^4^ MESs/mL (3.5 × 10^4^–3.5 × 10^5^ MESs/mL)) as well as HMC (average difference between donations: 2.5 × 10^4^ HMCs/mL (0–1.1 × 10^5^ HMCs/mL)) and MFG (average difference between donations: 11 × 10^4^ MFGs/mL (3.2 × 10^4^–3.4 × 10^5^ MFGs/mL)) concentration of samples. We noted a positive correlation between MES, MFG and HMC content. Microscopic analysis also confirmed observations that while the majority of MESs contain structures staining positively for Nile red, only a subset displayed a nucleus. Indeed, we found by this method that overall only 10.2% of pelleted structures represented nucleated cells, yielding a final concentration of approximately 1.2 × 10^4^ HMCs/mL (Fig. 1B, C). On the other hand, MFGs were present in an average concentration of 10.5 × 10^4^ MFGs/mL of milk, thereby representing 89.7% of all MESs (Fig. 1B, C, Fig. 2D, E). Together these findings highlight and support low HMC content amongst milk MESs and illustrate HMC heterogeneity.

### Microscopic Examination of Human Milk Cells and Milk Fat Globules Reveals Heterogeneity amongst Membrane Enclosed Structures and the Presence of Binucleated Milk Cells

**Fig. 3:**
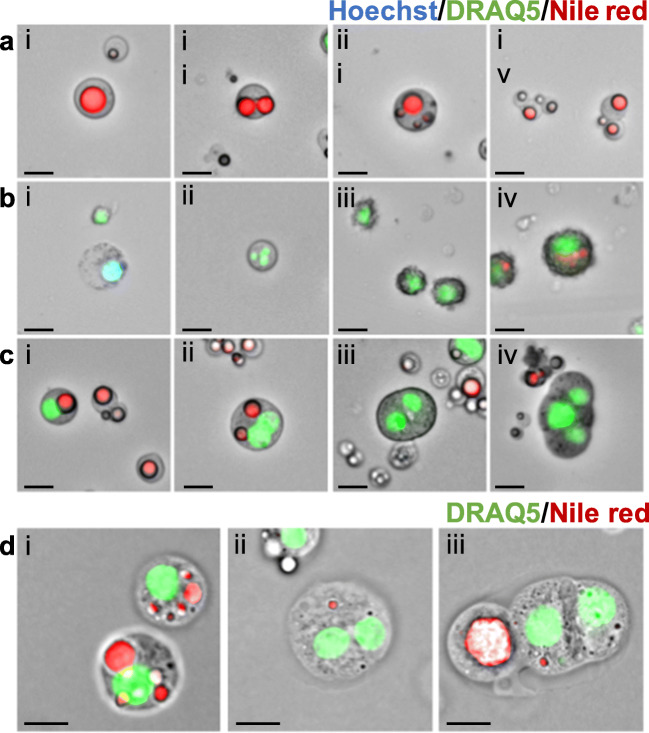
**Milk fat globule (MFG) and human milk cell (HMC) morphological variety**. A) MFGs show large variation both in size and morphology, as well as differences in the numbers of enclosed cytoplasmic lipid droplets (CLDs): i) MFG with large, single CLD, ii) MFG with two large CLDs, iii) MFG with one large and many small CLDs, iv) group of small MFGs. B) HMCs with immune cell-like morphology: i) myeloid precursor-like cell, ii) granulocyte-like cell, iii) lymphocyte-like cells, iv) dendritic cell-like morphology. C) standard cell-morphologies: i) with one single nucleus and a clearly visible CLDs, ii) binucleated cell, iii) two clustered cells and iv) a series of clustered cells. D) from left to right: i) cells showing numerous lipid droplets in the cytoplasm, ii) binucleated cell and iii) attached cells with budding MFG.

As described, the vast majority of MESs were non-nucleated, containing at least one or more cytoplasmic lipid droplets (CLDs) encircled by a membrane (visible as a black line, Fig. 3A) and staining positive for neutral lipid stain Nile red (Fig. 3A). These structures deviate from previously described MFG structure [18, 19] as a majority contained a significant amount of cytoplasm and were not necessarily limited to a single triglyceride core (Fig. 3A). Only a minor number of Nile red-positive structures were cytoplasm-free, observable exclusively on a different microscopic focal plane to the rest of the larger MESs (data not shown). MFGs showed enormous variation and were observed to reach similar sizes to the HMCs (MFGs: < 1.5–18.9 μm, HMCs: 4–22.6 μm). Additionally, CLDs were not limited to MFGs but were also found in HMCs (Fig.1, Fig. 2B, E, Fig. 3C, D), suggesting secretory activity [25–29].

As was observed by flow cytometric analysis, only a minor number of nucleated structures stained preferentially for Hoechst 33258 (Fig. 1A, C). These cells displayed a characteristic round and granular morphology resembling myeloid precursor cells (Fig. 3Bi), previously described to be found in human milk [30]. A considerable share of DRAQ5™^+^ cells also had morphologies resembling different immune cell subtypes thought to be present in human milk in fluctuating proportions dependent on the stage of lactation and health status of the mother/infant dyad [8, 30–32]. These cells including segmented nuclei as often found in granulocytes (Fig. 3Bii), lymphocyte-like smaller cells with visible protrusions (Fig. 3Biii) and large, rough-surfaced cells resembling dendritic cells (Fig. 3Biv). We did not examine these cells with specific immune cell markers, which may be considered a limitation of this study. That being said, a majority of cells displayed the nucleus situated closer to one side of the cell membrane alongside CLDs of varying number and size, matching the morphology of previously reported epithelial secretory luminal cells (Fig. 3C, D, E) [16, 25]. Cell clusters and strikingly binucleated cells were also commonly observed within the isolated pellet of most participant samples (Fig. 3Cii-iv, Dii-iii, Ei-ii). Whilst in some cases two or more cells appeared closely attached to one another (Fig. 3Cii, Dii, Eii), high powered imaging (630X) of HMCs embed in agarpads and stained with DRAQ5™ and Nile red, demonstrated the existence of binucleated cells in human milk at different stages of lactation (Fig. 3Dii, Eii).

**Fig. 4:**
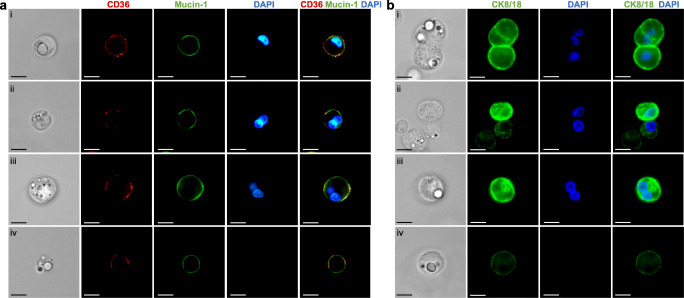
**Immunofluorescence images showing localisation and distribution of secretory and luminal markers across different membrane enclosed structures.** A) Staining of membrane bound proteins CD36 and Mucin-1 in representative images of i) mono-nucleated cells, ii-iii) binucleated cells and iv) non-nucleated milk fat globules (MFGs). B) CK8/18 staining in representative images of i) mononucleated and, ii) binucleated human milk cells (HMCs) iii) DAPI+ nucleated HMC clusters and iv) non-nucleated MFGs. Scale bars: 10 μm

To further characterize the structures found in the pelleted fraction of milk, we performed immunofluorescence imaging on fixed, stained and agar-embedded structures. We found that many of the membranes enclosing these structures stained positive for milk fat globule membrane associated glycoproteins CD36 and Mucin-1 (Fig. 4A) [19]. Among CD36^+^/Mucin-1^+^ MESs, we found mono- and bi-nucleated secretory cells (Fig. 4Ai-iii), as well as secreted non-nucleated MFGs (Fig. 4Aiv). To confirm the presence of luminal-lineage secretory cells in human milk we further stained the MES for cytokeratin 8/18 (CK8/18) (Fig. 4B). We identified CK8/18^+^ cell clusters, mono-nucleated and binucleated cells, as well as MFGs with low levels of CK8/18 expression (Fig. 4B). Here, we only examined a select number of lineage markers and hence did not characterize all cell types found in milk (such as immune). However, our results highlight that inventive fluorescence microscopy approaches clearly are a powerful tool to analyze HMCs and may be used to further investigate cell phenotype and protein expression in future studies.

### Milk Fat Globules Can be Eliminated by Freezing and Filtering

Due to the high concentration of MFGs in milk, accurate characterization and quantification of HMCs may be impaired. MFGs are directly secreted by HMCs (specifically secretory epithelial cells within the alveoli of the lactating mammary gland) and often contain HMC-derived cytoplasm, membrane proteins of different cellular origin, mRNA and organelles [6, 15, 17, 21, 27, 33–35], making it difficult to molecularly distinguish between the MESs. Despite these limitations, it still remains necessary to purify and quantify viable single cells from human milk, to effectively conduct single cell analysis or quantify colony forming ability of HMCs. As DRAQ5™ has previously been shown to impair cell proliferation by hindering cell cycle progression causing an arrest in G2 phase [36] fluorescence activated cell sorting was excluded, as unsuited for future culture experiments. We thereby set out to test alternative approaches to selectively separate HMCs from MFGs.

**Fig. 5:**
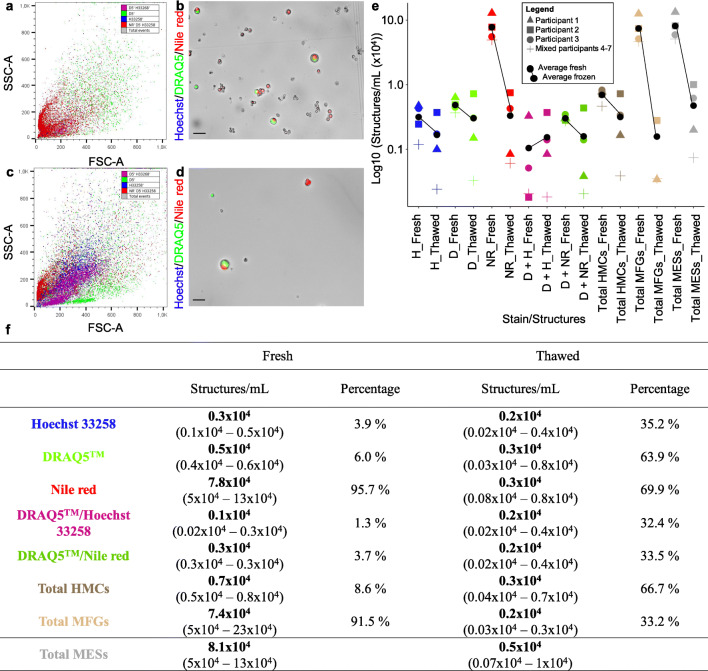
**Membrane enclosed structure (MES) population comparison before and after freezing on the same samples (*n*** **= 4)**. A) Representative flow cytometry plot showing distribution of nucleated and non-nucleated structures in fresh milk, according to granularity and size; high amount of red structures indicates strong milk fat globule (MFG) prevalence. B) Microscopic image of MESs found in freshly donated milk. Scale bar: 25 μm. C) Representative flow cytometry plot showing distribution of nucleated and non-nucleated structures contained in thawed milk; clear cell populations can be observed, indicating a decline of MFGs. D) Microscopic image of MESs found in thawed milk; most structures show nuclear staining while MFGs clearly diminished. Scale bar: 25 μm. E) Logarithmic representation of numerical differences between fresh and thawed MESs per mL of milk; geometrical shapes indicate individual donors, black dots and lines show overall average and trend. F) Average number and range of MESs, human milk cell (HMC) and MFG content of four samples counted in immunofluorescence and bright field images according to their staining pattern before and after thawing and corresponding percentages. Abbreviations: H: Hoechst 33258; D: DRAQ5^TM^; NR: Nile red

Preliminary observations in our laboratory found that numbers of MFGs were diminished following viable freezing of human milk MESs (unpublished). Optical analysis of thawed samples stained with Hoechst 33258, DRAQ5™ and Nile red clearly showed that MFG concentration was diminished following freezing procedure (Fig. 5B, D). Structures staining exclusively for Nile red were in fact significantly reduced (from 91.5% to 33.2%, *P* = 0.03; Fig. 5E, F), whereas nucleated cells appeared to be less susceptible to damage by freezing, consequently increasing from 8.6% of MESs to 66.7% after thawing (*P* = 0.07; Fig. 5E, F). Accordingly, a significant MFG decline from 88.8% to 55.7% (*P* = 0.03) was detected by flow cytometry of the same samples, while HMCs rose from 11.2% to 44.3% (*P* = 0.06) (see representative FSC/SSC dot plots Fig. 5A, C). The increased concentration of nucleated structures in the samples noticeably allowed for areas with a higher density of cells on the FSC vs. SSC plots to be distinguished (Fig. 5C). Interestingly, in contrast to our findings above (Fig. 1B, C), thawed samples displayed an increased number of MES that stained positive for both DRAQ5™ and Hoechst 33258, increasing the percentage of MESs stained with both dyes from 1.3% to 32.4% (Fig. 5E, F). Consequently, an increase from 1.1% to 25.9% of structures staining positive for DRAQ5™ and Hoechst 33258 was registered by flow cytometry (Fig. 5A, C). Overall, we observed a decline of all structures (from 8.1 × 10^4^ MESs/mL to 0.5 × 10^4^ MESs/mL), including both MFGs (from 7.4 × 10^4^ MFGs/mL to 0.2 × 10^4^ MFGs/mL) and HMCs (from 0.7 × 10^4^ HMCs/mL to 0.3 × 10^4^ HMCs/mL) (Fig. 5B, D-F) post-freezing.

**Fig. 6:**
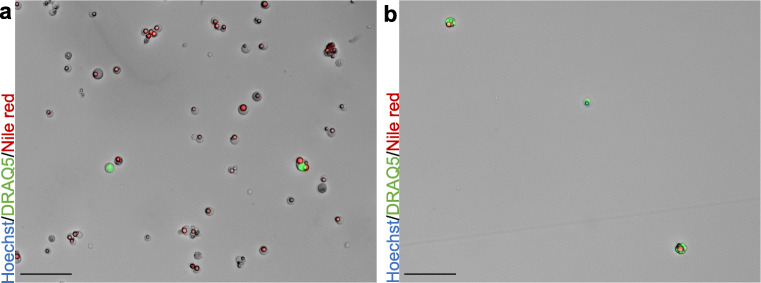
MACS® column filtration for milk fat globule (MFG) removal. A) Sample prior to filtration showing both MFGs and human milk cells (HMCs). B) Sample after filtration showing HMCs only. Scale bars: 50 μm

In addition to freezing, elimination of MFGs through MACS® column filtration was tested (*n* = 6). Fresh or thawed MESs were processed through the MACS® filtration system according to the supplied protocol. Subsequent staining of structures gathered in the flow-through with Hoechst 33258, DRAQ5™ and Nile red, showed that MFGs were completely absent in the filtered pellet (Fig. 6) and had thereby been retained inside the column. Nevertheless, cell numbers were drastically reduced as well, ranging between 0.5 and 2.8% of original MES for fresh samples and 7.1 to 18.1% in frozen samples. In total, between 5 and 27.6% of the total expected HMCs were retrieved.

## Discussion

Many studies investigating the composition of HMCs describe isolation methods using centrifugation to separate MFGs from HMCs (thought to exist in the upper fat layer and pellet, respectively), taking advantage of perceived discrepancies in their densities and size. A previous study found that cells may infiltrate the milk fat stratum [35] and another more recent publication suggested that MFGs may contaminate the milk cell pellet [17]. Here we confirmed recent findings showing that only a minority of isolated MESs are HMCs and go further to investigate the morphology of these MESs, as well as exploring possibilities in how they can be purified. We find, using nuclear stains examined with flow cytometry and fluorescence microscopy, that depending on the method, only 8.3 to 10.2% of MESs are nucleated cells. Through microscopic analysis we show that CK8/18^+^ epithelial HMCs vary in morphology and include not only cell clusters but also binucleated cells. Furthermore, we find that HMC and MFG separation is a challenging task and that aside from DRAQ5™ flow cytometric analysis, viably freezing, and MACS© filtering MESs allow for HMC enrichment.

Our study revealed that centrifuge-isolated HMCs and MFGs appear to show similarities across a number of features including size, lipid content, density and granularity. While previous studies have reported that on average only 7.2% (range 1–29%) of human MFGs display a cytoplasmic crescent [18, 21], we find that the majority of pelleted MFGs display a significant cytoplasmic content, suggesting that they are enriched following centrifugation. On a morphological level, varying numbers of Nile red^+^ triglyceride droplets were observed both in isolated cells and MFGs (Fig. 3A, C, D). This observation underlines the complexity of the fat globule budding process, in which MFGs as big as secretory HMCs are evidently routinely produced. As part of this process, a large amount of the cell’s cytoplasm is transferred to the newly secreted MFG in the form of the cytoplasmic crescent containing additional lipid droplets as well as cell organelles [6, 15, 21, 27]. As a consequence of this process, as illustrated by our study (**Figs.** **1****,** **2** and **3**), it becomes clear that on a microscopic level only the presence or absence of nuclei indicates whether an examined MES is a HMC or a MFG. Using both flow cytometry and fluorescent microscopy combined with bright field imaging, we show that on average only 8.1 to10.2% of structures resembling HMCs are indeed nucleated (Fig. 1). For previously published studies quantifying HMCs using bright-field or flow cytometry, these findings raise the question as to whether the number of cells in milk may be greatly overestimated, as MFGs have been shown to maintain proteins present on the membrane of the cell of origin [17, 27] as shown in this study (Fig. 4A). Overall, we estimate that human milk contains on average 1.2 × 10^4^ cells/mL (Fig. 1B, C), thereby on the low end of the previously reported range of 1 × 10^4^ to 13.5 × 10^6^ cells/mL [7]. Former studies found a strong correlation between fat content and cellular concentration in human milk [9, 32]. However, without the use of a nuclear stain to verify the nature of sequestered structures, it may be that the number of isolated MFGs increases in lipid rich milk, influencing the conclusion. Combining results obtained both through flow cytometrical analysis and various fluorescence microscopy approaches, we show that that only a fraction of MESs isolated from human milk by centrifugation are in fact nucleated, where incorporating nuclear stains into study design is vital for accurate visual HMC characterization or quantification.

Morphological variation of HMCs found by this study, including multi-nucleated and clustering cells, informs our understanding of the complexity of the human mammary gland during lactation. Binucleated cells arise due to cytokinetic failure, existing in high numbers (~30%) within the tissue of the lactating mammary gland and are evolutionarily conserved across a variety of different species [37]. These polyploid cells are thought to play an essential role during lactogenesis, assuring copious milk production by producing high amounts of milk protein [37], however binucleated cells have only once been described to be present in human ante- and early post-partum milk itself [20]. Here, we report for the first time that binucleated CD36^+^/Mucin-1^+^ secretory and CK8/18^+^ luminal cells are also released into human milk alongside other previously reported cell types at later stages of lactation, up to 62 weeks post-partum (Fig. 3). How these binucleated cells enter into the milk is unknown, however they may provide an excellent opportunity to more closely examine the role binucleated luminal epithelial cells may play in milk production and secretion.

Cell clusters present in human milk have been previously reported, when de novo formed tight junctions and interdigitations were observed connecting the cells [16, 20]. In our study we also observed a variety of cell clusters, with some aggregates consisting of three or more loosely attached cells and a majority consisting of two closely connected homogenous cells, as has been previously reported [16]. Many of these aggregated cells contained hallmarks of secretory epithelial alveolar cells of the lactating mammary gland such as CLDs or budding MFGs (Fig. 3Diii). These findings contradict previous theories that a majority of HMCs are of ductal origin, as CLDs are only rarely found in this cell type [16, 28, 29]. Whilst it remains unclear how and why secretory luminal cells enter human milk, it is interesting that many of the cells can be isolated mid-function, some even displaying budding MFGs (Fig. 3Diii). A limitation of this study is that we did not profile all milk mammary cell subtypes, such as immune cells, and hence future studies should focus on examining the protein and transcriptomic profile of these cells to better understand lactating mammary gland function and cell secretion.

Despite the necessity of developing reliable approaches to divide MFGs from live HMCs, this task proves more challenging than expected. In the course of this study, we examined different possibilities of separating HMCs from MFGs using MACS® dead cell removal kit or viable cryopreservation. When MACS® columns (which bind both apoptotic cells and cell fragments) were used to filter human milk MESs, fluorescence microscopy revealed the flow-through contained a very high purity of nucleated HMCs efficiently eliminating MFGs. However, recovered live HMCs ranged from 5 to 27.6% of total expected HMCs, thereby lower than the previously reported number of live cells per sample (8 to 81% of HMCs) [17]. Further analysis should thereby assess whether any unaccounted live cells are retained inside of the column. The method we found to be most successful in reducing the number of MFGs and recovering the most HMCs was through freezing and subsequent analysis of cryopreserved milk MESs samples. Post-thawing, a significant decrease in MFG concentration was observed, where MFGs were reduced from 91.5% to 33.2% of MESs (Fig. 4E, F). Previous studies have found that freezing human milk decreases the overall triglyceride concentration whilst increasing the concentration of free fatty acids, which, together with our results, suggests probable MFG membrane disruption and released lipids degradation by free lipases [38, 39]. In contrast, according to optical quantification, HMCs rose from 8.6% to 66.7% of total MES, although the absolute numbers of MESs decreased after freezing (Fig. 4E, F). Interestingly, the percentage of cells staining for both DRAQ5™ and Hoechst 33258 increased as well (from 1.3% to 32.4%, Fig. 4E, F) potentially indicating higher numbers of senescent cells with a damaged membrane facilitating dye uptake. Taken together, we illustrate that separating MFGs from HMCs is not simple and should be improved in the future, perhaps with the use of solvents, to better study these cells.

Cells isolated from human milk provide an opportunity to examine and characterize the lactating mammary epithelium. Whilst cells have been historically reported to be simple to isolate from the milk through centrifugation, this study confirms recent findings that only a minority of cell-like structures isolated contain a nucleus. The present study underlines the importance and complexity of separating HMCs from MFGs due to the similarity of these structures in size, granularity and lipid content. We additionally show that the variety of different epithelial cell types, such as secretory binucleated epithelial cells, are present in milk mirroring the complexity of the human mammary gland during lactation. This study provides novel methods and insights for studying lactating cells and paves the way for future studies to understand the characteristics, functions and potential implications of cells in human milk.
